# Gelatinase Responsive Nanogel for Antibacterial Phototherapy and Wound Healing

**DOI:** 10.3390/gels8070397

**Published:** 2022-06-23

**Authors:** Qianqian Han, Xuan Wang, Lin Qiu, Xinpei Zhou, Zexuan Hui, Xinye Ni, Yang Xuan, Xiaoling Lei, Jianhao Wang

**Affiliations:** 1 School of Pharmacy, Changzhou University, Changzhou 213164, China; 200817z1018@smail.cczu.edu.cn (Q.H.); 19085235788@smail.cczu.edu.cn (X.W.); linqiu@cczu.edu.cn (L.Q.); 19479125@smail.cczu.edu.cn (X.Z.); 19479127@smail.cczu.edu.cn (Z.H.); 2 The Affiliated Changzhou No. 2 People’s Hospital of Nanjing Medical University, Changzhou 213003, China; 3 Key Lab of Biotechnology and Bioresources Utilization of Ministry of Education, College of Life Science, Dalian Minzu University, Dalian 116600, China; xuanyang@dlnu.edu.cn; 4 College of Life Science and Technology, Huazhong University of Science and Technology, Wuhan 430074, China; d202180741@hust.edu.cn

**Keywords:** CuS NDs, gelatin nanogel, targeted drug release, antibacterial activity, wound healing

## Abstract

The unique bactericidal mechanism of metal nanoparticles (MNPs) is considered to be an effective strategy to deal with antibiotic resistance, but the oxidative stress damage caused by excessive accumulation of MNPs to normal cells cannot be ignored. Achieving on-demand release of nano-drugs in specific infection environments is highly attractive. Herein, we constructed a “core-shell” nanogel (G@CuS) based on a copper sulfide (CuS) antimicrobial agent and gelatin for targeted drug release and bacterial clearance in a gelatinase infected microenvironment. G@CuS produced heat and reactive oxygen species (ROS) under the irradiation of a laser, which together with the released Cu^2+^ cause irreversible and efficient physical damage to the bacteria. Moreover, the encapsulation of gelatin not only limits the biotoxicity of CuS nanodots (NDs), but also effectively promotes the proliferation of mammalian cells. Under the synergy of multiple mechanisms, G@CuS eradicated the colonized bacteria in the wound of mice infected with *Staphylococcus aureus* (*S. aureus*) and accelerated wound healing. The proposed application strategy of nanogel is expected to provide a new idea for clinical transformation.

## 1. Introduction

As the largest organ of the human body, the skin has many functions, such as maintaining metabolic homeostasis, regulating body fluid balance, and preventing microbial invasion. Due to direct contact with the outside world, the skin is highly susceptible to open damage [1,2,3]. Most injuries can be repaired autonomously through complex biological events, while chronic wounds, such as those caused by bacterial infections, are difficult to restore to their ideal state in a short period of time [4,5,6]. Antibiotics are still the main tool in the clinical treatment of bacterial infections, but the successive arrival of various drug-resistant bacteria gradually weakens its efficacy [7,8]. Finding novel strategies to replace antibiotic therapy has become an urgent need of modern medicine.

Actually, many strategies have been developed to help cast off the medical dilemma caused by drug-resistant bacteria at this stage, including antibacterial nanoparticles [9,10], antimicrobial peptides [11,12], and photothermal/photodynamic therapy (PTT/PDT). Among them, PTT employs light source to induce photothermal agents (PATs) to generate heat, thereby increasing the local temperature and leading to the thermal ablation and apoptosis of bacteria. Meanwhile, the concept of PDT is that photosensitizers (PSs) transmit photon energy to produce reactive oxygen species (ROS) under the irradiation of light, thus disrupting the normal physiological metabolic activities of bacterial cells and inactivating them [13,14,15,16]. Recently, some studies have attempted to combine these two phototherapy strategies in order to achieve better therapeutic effects. However, most synergistic phototherapy strategies require the integration of different phototherapy agents or even two-wavelength laser irradiation, resulting in superimposed side effects and delayed treatment [17,18]. Therefore, it is very attractive to construct single-wavelength-activated PTT/PDT-integrated photoresponse materials. As a typical p-type semiconductor, copper sulfide (CuS) can not only perform efficient photothermal conversion through surface plasmon resonance (SPR), but also produce ROS under laser irradiation, showing fascinating phototherapy potential [19,20,21,22]. In addition, the antibacterial activity of copper-based nanomaterials has been proved to be related to the release of Cu^2+^, which destroys the integrity of the bacterial cell structure by solidifying proteins or disrupting enzyme function [23,24]. On the other hand, the peroxidation produced during the conversion of Cu^2+^ and Cu^+^ is also considered to be an important factor in killing pathogens [25]. However, oxidative stress caused by the excessive release of Cu^2+^ will also indiscriminately damage normal tissue cells, resulting in unpredictable toxicity problems [26,27,28,29]. Therefore, the development of an on-demand drug release platform in response to the infection microenvironment is expected to improve the biosafety of CuS.

Matrix metalloproteinases (MMPs), especially gelatinases (MMP-2 and MMP-9), are markers of *Staphylococcus aureus* (*S. aureus*) infection sites [30]. As a hydrolyzed product of collagen, gelatin can be degraded into small biomolecules by collagenase and has reliable biological safety, and thus is widely used as a drug carrier for the treatment of bacterial infections [31,32,33,34]. For instance, Li et al. reported an antibiotic delivery system based on gelatin nanoparticles, which utilizes the degradation of gelatin by gelatinase to release encapsulated vancomycin in situ at the infection site to kill pathogenic bacteria, avoiding systemic toxicity from overuse of antibiotics [35]. Furthermore, gelatin can also effectively promote wound healing, which has been confirmed in previous work [36,37].

**Scheme 1:**
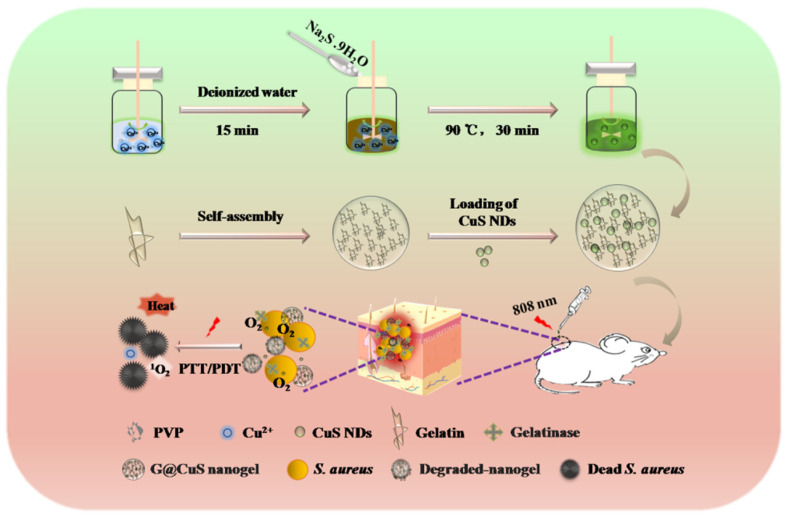
Schematic illustration of the application of the G@CuS in the treatment of *S. aureus* infected wounds.

Considering all these in mind, a “core–shell” nanogel (G@CuS) based on CuS nanodots (NDs) and gelatin nanogel (Gel) was constructed by the desolvation method and electrostatic interaction in this study. G@CuS can release Cu^2+^ in situ only in the presence of gelatinase and cause irreversible damage to bacteria by the ROS and heat generated under the same light source (808 nm) irradiation. Therefore, the encapsulation of gelatin can not only effectively improve the biocompatibility and stability of CuS NDs, but also not limit their phototherapeutic properties. In the *S. aureus*-infected mice model, the combination of the above antibacterial mechanisms can effectively eradicate bacteria on the wound surface and accelerate wound healing (Scheme 1).

## 2. Results and Discussion

### 2.1. Preparation and Characterization of G@CuS

**Figure 1:**
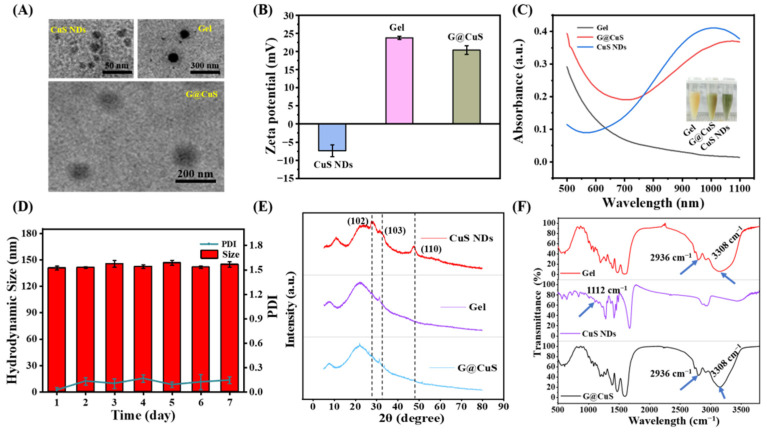
Characterization of CuS NDs, Gel, and G@CuS: (**A**) TEM images of CuS NDs, Gel, and G@CuS; (**B**) ζ-potential of CuS NDs, Gel, and G@CuS; (**C**) UV-vis spectra and pictures of Gel, G@CuS, and CuS NDs; (**D**) 7-day particle size and PDI changes of the G@CuS; (**E**) XRD pattern of standard CuS NDs, Gel, and G@CuS; (**F**) FT-IR spectra of Gel, CuS NDs, and G@CuS. (*n* = 3).

CuS NDs was synthesized by a simple hydrothermal method and entrapped into degummed Gel to form G@CuS nanogel. The encapsulation efficiency determined by centrifugation demonstrated that this formulation method was stable and satisfactory (Figure S1). The morphology and size distribution of the G@CuS nanogels were studied by TEM and DLS. CuS NDs were dispersed point-like particles, and the Gel was smooth microspheres with uniform size, whereas the Gel loaded with CuS NDs was rough in shape and had fuzzy edges, showing a typical “core–shell” structure, indicating the successful synthesis of G@CuS nanogel. In addition, the TEM images showed that the sizes of CuS NDs, Gel and G@CuS were ~10 nm, ~120 nm and ~145 nm, respectively, which were roughly the same as those measured by DLS (Figure 1A and Figure S2). Meanwhile, the ζ-potentials of the CuS NDs and Gel were approximately −7.4 and +23.7 mV, respectively, and became +20.4 mV after combination. This change was attributed to the charge neutralization caused by electrostatic interaction (Figure 1B). UV-vis spectra showed that G@CuS had similar absorption spectra to CuS NDs in the NIR range of 750–1100 nm, indicating the potential photothermal conversion performance of G@CuS (Figure 1C). It is worth noting that the particle size of G@CuS dispersion system was stable during 1 week of storage at 4 °C, showing good storability (Figure 1D). The diffraction peaks in the XRD pattern of CuS NDs were consistent with the standard card (00001-1281). The diffraction peaks located at 2θ of 29.04, 31.96, and 47.86 were attributed to (102), (103), and (110) planes, respectively, but there were no corresponding diffraction peaks in G@CuS, indicating that CuS NDs synthesis was correct and that it was effectively encapsulated by the Gel (Figure 1E). Furthermore, the only characteristic peaks at 2936 cm^−1^ and 3308 cm^−1^, which corresponded to C-H and O-H stretching modes, respectively, appeared in the FT-IR spectra of G@CuS, and no characteristic peaks of new covalent bonds were detected, indicating that there is only a simple physical relationship between gelatin and CuS NDs. The disappearance of the characteristic peak belonging to CuS NDs at 1112 cm^−^^1^ was attributed to the dilution effect of the excipients [38,39] (Figure 1F).

### 2.2. Phototherapeutic Properties of G@CuS

**Figure 2:**
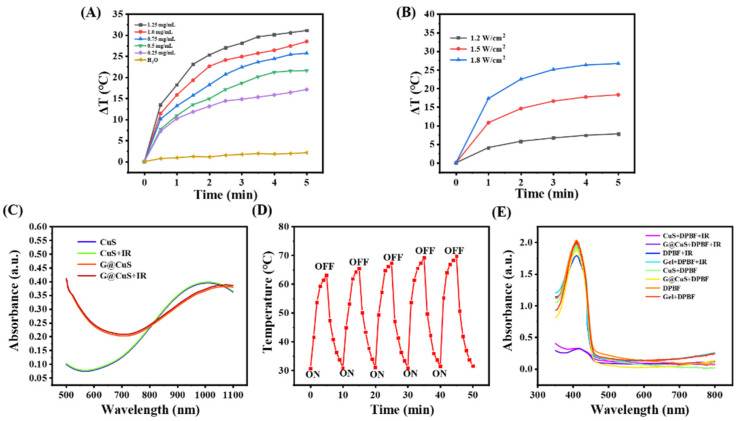
(**A**) Concentration-dependent temperature changes of the G@CuS under 808 nm laser irradiation; (**B**) Power-dependent temperature changes of the G@CuS (0.75 mg mL^−1^) under 808 nm laser irradiation; (**C**) Changes of absorption spectra of CuS NDs and G@CuS before and after NIR laser (808 nm, 1.8 W cm^−2^, 5 min); (**D**) Temperature changes of the G@CuS (1.25 mg mL^−1^) during five on/off cycles of NIR laser (808 nm, 1.8 W cm^−2^) irradiation; (**E**) UV-vis spectra of DPBF before and after adding CuS NDs, G@CuS (0.75 mg mL^−1^), and NIR irradiation (808 nm, 1.8 W cm^−2^).

CuS NDs have been proved to be an excellent phototherapeutic agent in previous studies [3]. To evaluate whether the packaging of the Gel will affect the phototherapeutic properties of the CuS NDs, the photothermal behavior of G@CuS exposed to NIR laser (808 nm, 1.8 W cm^−2^) was studied. As shown in Figure 2A and Figure S3, per the benefit from the strong penetration ability of NIR, the G@CuS had a rapid heating effect in a concentration-dependent manner, and a steady temperature peak could be reached after 5 min. Moreover, the photothermal effect of the G@CuS would become more obvious with the increase of laser power (Figure 2B). Moreover, as that of CuS NDs, the UV-vis absorption of the G@CuS irradiated by the NIR laser (808 nm, 1.8 W cm^−2^, 5 min) was not significantly decreased, which proved that there were no photobleaching defects as fluorescent photothermal agents (Figure 2C). The stable heating effect of the G@CuS in the laser on/off five times also supported this view, and its photothermal effect would not change for a long time (Figure 2D). Using DPBF as an indicator of ^1^O_2_ production, the effect of the Gel encapsulation on the ROS release was measured. As shown in Figure 2E, the UV-vis absorption of DPBF coincubated with the G@CuS decreased significantly after the NIR laser (808 nm, 1.8 W cm^−2^, 5 min) irradiation, which proved that ^1^O_2_ could escape from the package of the Gel. Taken together, the G@CuS still retained the phototherapeutic potential of the CuS NDs.

### 2.3. In Vitro Antibacterial Activity

*S. aureus* was known to secrete type IV gelatinase to hydrolyze Gelatin. Herein, we used *E. coli* as the control strain to evaluate the targeted antibacterial ability of the G@CuS. Due to the release of Cu^2+^, the CuS NDs could effectively kill both kinds of bacteria and had broad-spectrum antibacterial activity. In particular, E. coli showed more intolerance, and the exact reason was not clear (Figure S4). The amount of packaging of the Gel to the CuS NDs was determined by the standard curve of the CuS NDs (Figure S5). Different concentrations of free CuS NDs were encapsulated in the Gel and reincubated with two kinds of bacteria, and some interesting results were obtained. Due to the hydrolysis of Gelatin by gelatinase and the release of Cu^2+^ from the Gel, the effect of the G@CuS on *S. aureus* was about the same as that of the free CuS NDs. In contrast, even in the G@CuS environment of 1 mg mL^−1^, the survival rate of *E. coli* was still higher than 80%, and the effect was negligible (Figure S6).

**Figure 3:**
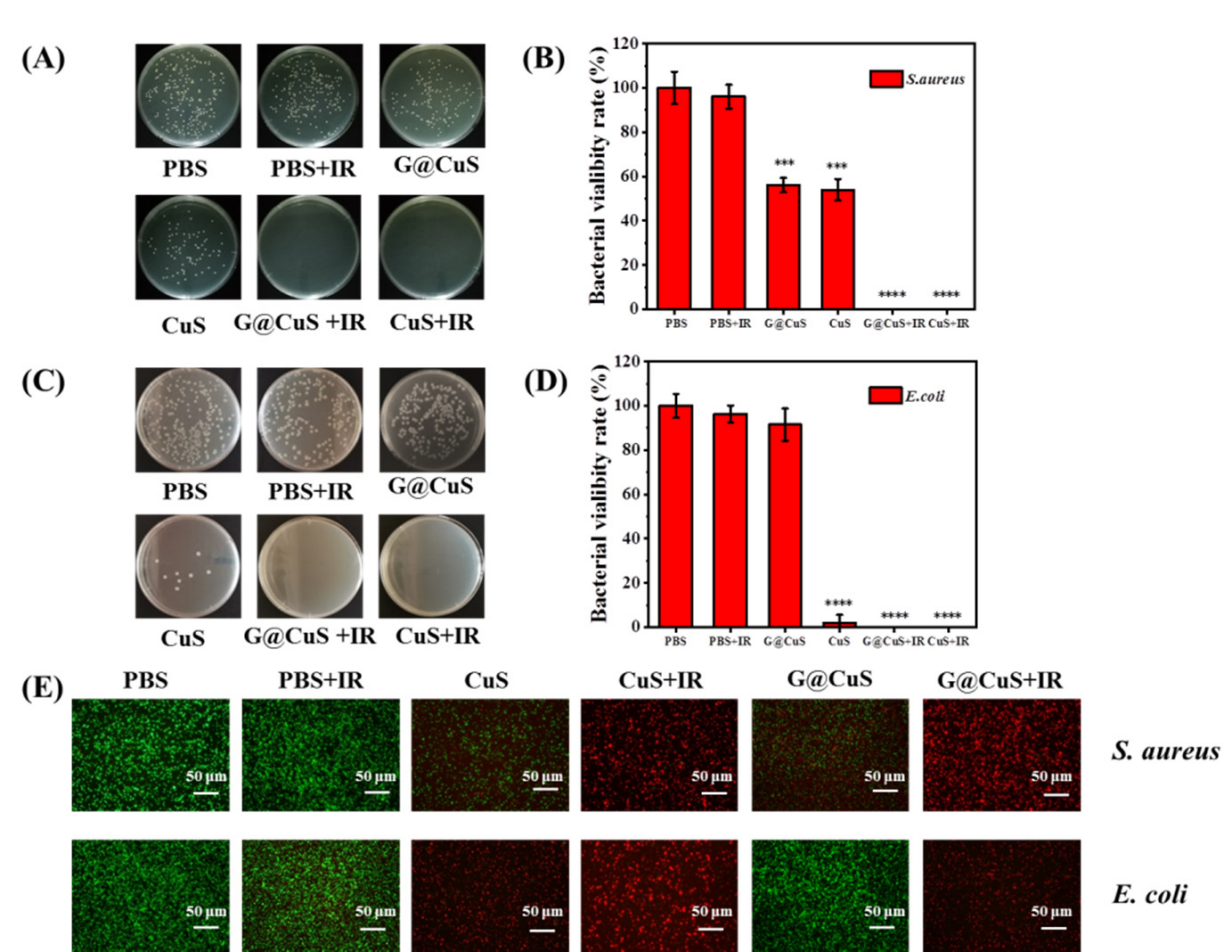
Colony images (**A**) and a statistical chart (**B**) of *S. aureus* incubated with CuS NDs and G@CuS before and after NIR laser (808 nm, 1.8 W cm^−2^, 5 min) irradiation; Colony images (**C**) and a statistical chart (**D**) of *E. coli* incubated with CuS NDs and G@CuS before and after NIR laser (808 nm, 1.8 W cm^−2^, 5 min) irradiation; (**E**) Representative overlapping fluorescence images for the live/dead staining assay of *S. aureus* and *E. coli* incubated with CuS NDs and G@CuS before and after NIR laser (808 nm, 1.8 W cm^−2^, 5 min) irradiation. (*n* = 3; *** *p* < 0.001, **** *p* < 0.0001).

Next, the NIR laser (808 nm, 1.8 W cm^−2^) was introduced on the basis of 0.75 mg mL^−1^ CuS NDs and the G@CuS to explore the synergistic antibacterial activity of phototherapy. As shown in Figure 3, bacteria in the PBS group showed roughly the same survival rate regardless of whether they were irradiated. However, the CuS NDs and G@CuS groups achieved the complete removal of bacteria when irradiated with the NIR laser, even the G@CuS, which had previously been less effective against *E. coli*.

Live/dead bacterial cell staining assay was used to further detect the antibacterial activity of each component. The bacterial cells with damaged cell membrane were stained with PI and emitted red fluorescence signal, while SYTO 9 could only label the living cells green. The bacteria in the PBS group emitted a strong green fluorescence signal, indicating that the bacterial cells had good activity. The groups of the G@CuS and CuS NDs assisted by the laser were all red fluorescence in the field of vision, showing more excellent germicidal properties. The germicidal efficacy of the other groups was relatively limited, which was basically the same as that of the agar plate (Figure 3E). In addition, crystal violet staining results showed that the laser-irradiated groups could not only effectively inhibit the formation of bacterial biofilm, but also promote the destruction of the integrated biofilm (Figure S7).

### 2.4. In Vitro Biocompatibility

**Figure 4:**
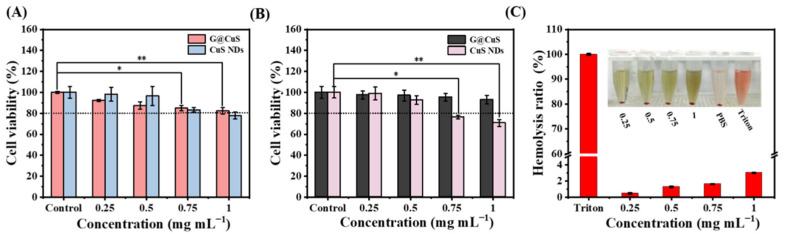
(**A**) Cytotoxicity of different concentrations of G@CuS and CuS NDs incubated with *HUVECs*; (**B**) Cytotoxicity of different concentrations of G@CuS and CuS NDs incubated with *L929s*; (**C**) Hemolysis ratio and image of red blood cells incubated with different concentrations of the G@CuS. (*n* = 3; * *p* < 0.05, ** *p* < 0.01).

Excessive accumulation of Cu^2+^ will lead to strong oxidative stress and potential toxicity. The biosafety of the G@CuS was evaluated by investigating its toxicity to mammalian cells and red blood cells. As shown in Figure 4A,B, in HUVECs and *L929s*, after incubated with 1 mg mL^−1^ of CuS NDs for 24 h, the cell viability was lower than 80%, showing significant cytotoxicity. Fortunately, after encapsulation by the Gel, the cytotoxicity of the CuS NDs was inhibited, making them more friendly to mammalian cells. Moreover, red blood cells cause free CuS NDs to accumulate and settle, which aroused our concern (Figure S8). Encouragingly, there was no such phenomenon in the coincubation of red blood cells with the G@CuS, and the hemolysis rate was always less than 5%, which further indicated that the encapsulation of the Gel effectively improved the biocompatibility of the CuS NDs (Figure 4C). Significant hemolysis and cytotoxicity were also not induced in the laser-irradiated G@CuS (Figure S9).

Considering that the Gelatin could promote cell adhesion and induce cell proliferation and differentiation, the effect of Gelatin on cell proliferation and migration was further evaluated on the premise of ensuring the good biological safety of the G@CuS. As shown in Figure S10, the G@CuS were incubated with *L929s* for 48 h, and UV-vis absorption quantification showed that, compared with the control group, the cell viability was more vigorous, indicating that the cells could effectively proliferate in the presence of the G@CuS. Furthermore, the phenomenon of cell proliferation and migration could be observed more intuitively through the cell scratch assay. With the passage of time (0-48 h), the scratch area became narrower in the G@CuS group, while only a few cells could be found in the control scratch area. These results showed that the G@CuS had great potential for wound healing.

### 2.5. Antibacterial Activity In Vivo and Wound Healing

**Figure 5:**
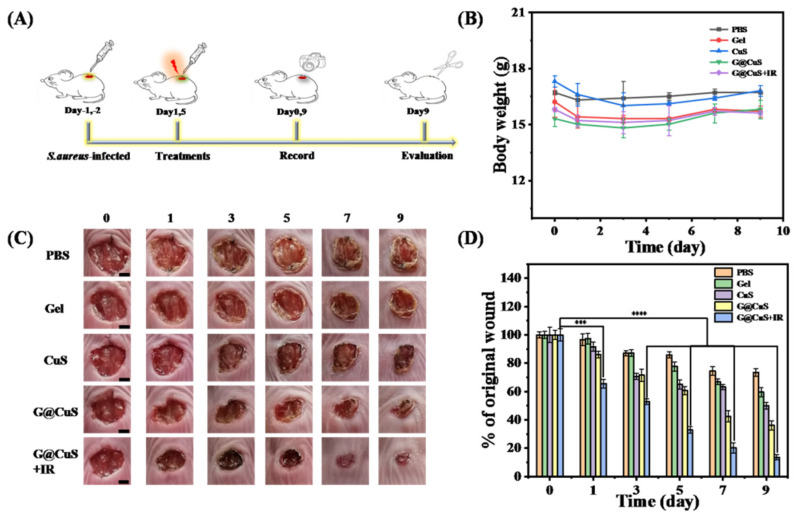
(**A**) Schematic illustration of the operation of wound establishment, treatment and examination; (**B**) Changes in weight of mice in each group for 9 days of treatment; (**C**) Representative pictures of wound tissue on different days for each treatment group. The scale bar represents 2 mm; (**D**) The relative sizes (% of the original wound) of *S. aureus*-infected wounds of each treatment group were compared every other day for 9 days. Data were expressed as standard deviations. (*n* = 3; *** *p* < 0.001, **** *p* < 0.0001).

Encouraged by the excellent antibacterial activity and the cell proliferation of G@CuS in vitro, we further used it in the treatment of S. aureus-infected wounds in vivo. Briefly, after the wounds were infected with *S. aureus*, each group of mice was then given different treatment (PBS, CuS, Gel, G@CuS, and G@CuS + IR) for 5 days. On the nineth day of treatment, the mice were killed, and the wound healing effect was evaluated based on histopathology (Figure 5A). During phototherapy, the wound skin temperature was always maintained at around 45 °C to avoid thermal damage to surrounding normal tissues. The weight of mice in each group changed steadily without obvious discomfort during the treatment (Figure 5B). Wound images were recorded every day and the healing areas were quantitatively calculated to monitor the wound healing effect. The results showed that the wounds of the CuS, G@CuS, and G@CuS + IR treatment groups all contracted on the third day, but there was no significant change in the PBS and Gel groups. On the nineth day, the wound of the G@CuS + IR group almost healed (the wound healing rate was about 87%), and only a small scar was left. Oppositely, the wound healing rate in the PBS, Gel, and CuS groups were 27%, 41%, and 51%, respectively, and the wounds were still covered with thick scars. Compared with these three groups, the healing situation of the G@CuS group was relatively better, and the scab had fallen off (the healing rate reached 62%) (Figure 5C,D).

**Figure 6:**
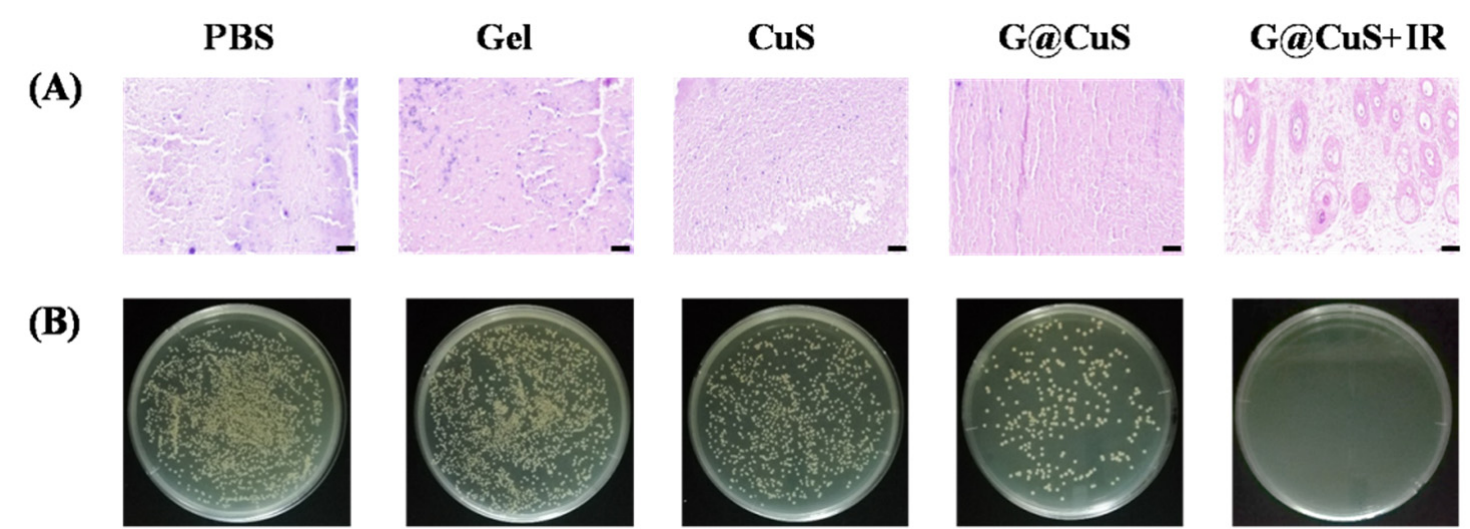
(**A**) Gram staining images of the skin tissue of mice infected with *S. aureus* in each treatment group treated for 9 days. The scale bar represents 50 μm; (**B**) The images of a plate colony in the skin of mice infected with *S. aureus* in each treatment group.

After nine days, the mice were sacrificed and dissected, and the skin wound tissue homogenate was plated on agar plates. As shown in Figure 6, there were still many colonies on the agar plates which were corresponding to the PBS and Gel groups, indicating that wounds were still infiltrated by bacteria. In contrast, the number of bacteria decreased in the CuS and G@CuS groups due to the effect of Cu^2+^, while *S. aureus* in the G@CuS + IR group was completely eradicated, which was further confirmed by Gram staining. These results suggested that bacterial clearance was positively correlated with wound healing efficacy.

**Figure 7:**
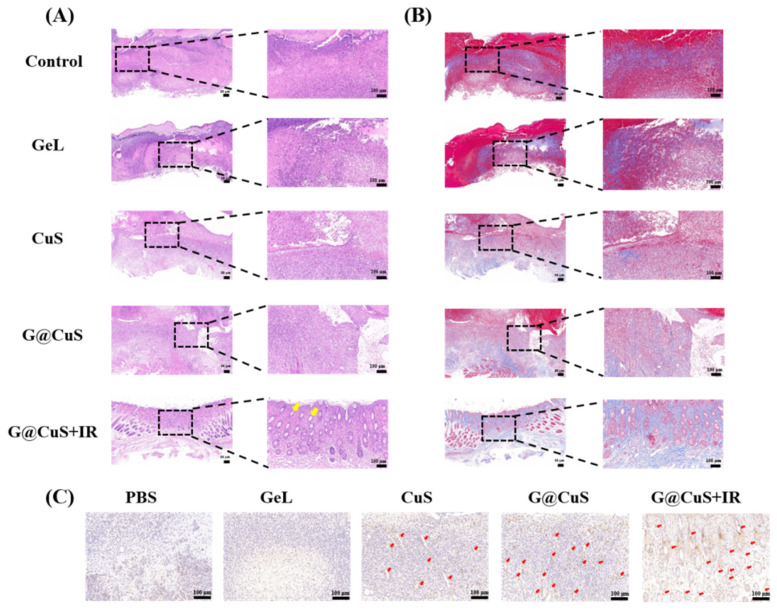
Images of (**A**) H&E staining, (**B**) Masson’s trichrome staining and (**C**) CD31 immunohistochemical in the regenerated tissues treated for 9 days.

Lastly, the wound-healing effect of each group was further evaluated by H&E staining, Masson’s trichrome staining, and CD31 immunohistochemistry. As shown in Figure 7A, the H&E staining showed that, after nine days of the G@CuS + IR treatment, the wound structure was flattened, the epidermis was continuous and dense, and new hair follicles appeared in the dermis (yellow arrow). On the contrary, the PBS, Gel, and CuS groups were infiltrated by a large number of inflammatory cells and granulation tissue was loose and ulcerated. The inflammatory reaction in the G@CuS group was mild, which could be explained by the mild removal of wound infection bacteria by the on-demand release of Cu^2+^, and the timely moisturization of new granulation tissue by gelatin. Masson staining showed that only the G@CuS + IR group formed thick and dense collagen fibers with regular orientation and topological structure. The wounds of the other groups were wrapped in scar tissue, with only a small amount of collagen deposition and sparse arrangement, showing a poor effect of regeneration (Figure 7B).

Similarly, the G@CuS + IR group showed more positive fine fruits in endothelial cell marker CD31 immunohistochemical staining, which indicated that the G@CuS + IR group had a huge vascularization trend, which provided more favorable conditions such as oxygen and nutrients for wound healing. Taken together, the G@CuS combined with multifaceted mechanism of action could effectively remove wound bacteria and promote tissue reconstruction (Figure 7C). Moreover, there were no pathological changes in the main organ structures in each group, which confirmed the safety and feasibility of our material (Figure S11).

## 3. Experimental Section

### 3.1. Materials

Copper (II) chloride dehydrate (CuCl_2_·2H_2_O), polyvinylpyrrolidone (PVP, M.W. 40000), sodium sulfide nonahydrate (Na_2_S·9H_2_O), and glutaraldehyde were purchased from Adamas-Beta. The 1, 3-diphenylisobenzofuran (DPBF), crystal violet stain solution, 3-(4,5-dimethylthiazol-2-yl)-2, 5-diphenyltetrazolium bromide (MTT reagent), phosphate-buffered saline (PBS buffer), Luria–Bertani broth (LB), and Oleic acid and dimethyl sulfoxide (DMSO) were obtained from Sigma-Aldrich. A live/dead bacteria viability kit, Dulbecco’s modified Eagle’s medium (DMEM), and fetal bovine serum (FBS) were purchased from ThermoFisher. Penicillin−streptomycin was purchased from Beyotime Biotechnology. A-type gelatin was obtained from Merck. Deionized (DI) water (Millipore Milli-Q grade, 18.2 MΩ) was used in all experiments. All chemicals used without further purification.

### 3.2. Synthesis of CuS NDs

The synthesis of CuS NDs was followed the previously published method with a slight modification [40]. Briefly, 21 mg CuCl_2_·2H_2_O and 60 mg PVP were added to 5 mL DI water at room temperature with constant stirring. After the mixture became a clear solution, 30 mg Na_2_S·9H_2_O was added to it and reacted at 90 °C for 30 min to obtain a dark green solution. CuS NDs can be obtained after 24 h of dialysis in DI water.

### 3.3. Preparation of G@CuS

The synthesis of the Gel was prepared by the previously published method [41]. Briefly, 0.55 g A-type Gelatin was added to a round-bottom flask, dissolved in 15 mL DI water, and 15 mL acetone was added with stirring. After the mixture stratified, the supernatant was discarded, and the precipitation was heated to dissolve in 7 mL DI water. Then, CuS NDs (8 mg mL^−1^, 3 mL) were slowly added dropwise to the solution and the pH was adjusted to 2.5 with 1 M HCl. An amount of 30 mL of acetone was added dropwise to the solution and became milky white gradually, and then 50% glutaraldehyde (40 μL) was added to continue stirring for 16 h to cross-link the Gelatin and form composite nanogel (G@CuS).

### 3.4. Characterization of G@CuS

The particle size and dispersion of Gel, CuS NDs, and G@CuS were measured using a Malvern particle size analyzer (Zetasizer Nano ZS90, Malvern, UK). The UV-vis spectra of the Gel, CuS NDs, and G@CuS were measured by ultraviolet spectrophotometer (UV-1900i, Shimadzu, Kyoto, Japan). The size and morphology of the Gel, CuS NDs, and G@CuS were studied using transmission electron microscopy (TEM, Tecnai G20, FEI, Hillsboro, OR, USA). The intermolecular force of the Gel, CuS NDs, and G@CuS were studied by Fourier transform infrared spectroscopy (FT-IR, Nicolet iS50, ThermoFisher, Waltham, MA, USA). The X-ray diffraction spectra were obtained using an X-ray diffractometer (XRD, Smartlab9, Rigaku, Kyoto, Japan).

### 3.5. Establishment of CuS NDs Standard Curve

CuS NDs aqueous solutions of different concentrations (0.25, 0.5, 0.75, 1.0, 1.25, 1.5 mg mL^−^^1^) were prepared, and the absorbance value of each sample at 1000 nm was measured using an ultraviolet spectrophotometer, and the average value was obtained after 5 parallel measurements. Correlation curves were made based on concentration and absorbance, and a linear fit was performed using Origin 2018 (OriginLab software, Northampton, MA, USA).

### 3.6. Determination of Encapsulation Efficiency

Encapsulation efficiency (EE) was indirectly determined by measuring the amount of free CuS NDs in the supernatant solution after centrifugation. G@CuS dispersion was centrifuged (13,000 rpm, 4 °C) for 20 min using a freeze centrifuge (H1850R, Cence, Changsha, China), and then the supernatant was collected to measure the free CuS NDs concentration by the standard curve of CuS NDs. The percent of encapsulated CuS NDs was calculated according to Equation (1): [42]

(1) $$\mathrm{EE}\%\;=\;\frac{W_{i}\;-W_{f}}{W_{i}}\;\times 100\%$$

where $W_{i}$ is the amount of initial CuS NDs added to the reaction system and $W_{f}$ is the amount of free CuS NDs detected in the supernatant solution after centrifugation. The result was the average value of the three measurements, expressed as the final concentration of CuS NDs in the system.

### 3.7. Photothermal Performance of G@CuS

To measure the photothermal behavior of G@CuS under the near-infrared (NIR) irradiation, 200 μL G@CuS (0, 0.25, 0.5, 0.75, 1.0 mg mL^−1^) were irradiated by NIR laser (808 nm, 1.8 W cm^−2^, 5 min), and the temperature was recorded every 30 s by thermal imager. G@CuS (0.75 mg mL^−1^) were then irradiated by 808 nm laser of different powers (1.2, 1.5, 1.8 W cm^−2^), and the temperature was recorded every 1 min. The photothermal stability of G@CuS was evaluated by laser on/off 5 times NIR irradiation (808 nm, 1.8 W cm^−2^, 5 min). The concentration of G@CuS was quantified based on the standard curve of CuS NDs unless otherwise noted.

### 3.8. Evaluation of Reactive Oxygen Species (ROS) Generation

The ability of G@CuS to produce ROS under NIR laser was detected by DPBF. In short, all samples were mixed into DPBF solution and irradiated by NIR laser (808 nm, 1.8 W cm^−2^, 5 min). The UV-vis absorption curve of DPBF was obtained by a microplate reader (Synergy NEO, BioTek, Winooski, VT, USA).

### 3.9. Bacterial Culture

*S. aureus* (ATCC 6538) and *E. coli* (ATCC 8739) were used for the antibacterial studies. The bacteria were incubated overnight in 10 mL LB medium in a shaker (37 °C, 250 rpm) and obtained at the exponential growth phase via centrifugation. The bacterial concentration could be monitored photometrically by measuring the optical density (OD) value at 600 nm. When the OD_600_ value was 0.1, the corresponding concentration of *S. aureus* and *E. coli* was 2 × 10^8^ and 4 × 10^8^ CFU mL^−1^, respectively.

### 3.10. In Vitro Antibacterial Activity of G@CuS

Different concentrations of G@CuS were incubated with *S. aureus* or *E. coli* (10^8^ CFU mL^−1^) at 37 °C for 1 h. The NIR laser (808 nm, 1.8 W cm^−2^) was used to irradiate 5 min in the irradiation groups. After incubation, the mixed bacterial solution was diluted properly and 100 μL solution was inoculated on agar plate, and then cultured overnight at 37 °C. The inhibitory effect of G@CuS on bacteria was systematically evaluated by counting bacterial colonies. In vitro experiments involving bacteria and cells used PBS as a negative control unless otherwise stated.

### 3.11. Live/Dead Bacterial Staining Assay

An amount of 0.75 mg mL^−1^ G@CuS and free CuS NDs were incubated with *S. aureus* or *E. coli* for 30 min, respectively. The NIR laser (808 nm, 1.8 W cm^−2^, 5 min) was used in the irradiation groups. The upper culture medium was removed by freezing centrifuge (4 °C, 5000 rpm, 5 min), and the precipitate was stained with SYTO 9/PI for 0.5 h in the dark. SYTO 9-labeled living cells showed green, while PI-labeled dead cells emitted red fluorescence. The survival of bacteria was observed by inverted fluorescence microscope.

### 3.12. Inhibition of the Biofilm Formation

The 100 μL bacterial solution (10^8^ CFU mL^−1^) and G@CuS were inoculated into a 96-well plate, and the irradiation group was irradiated with NIR laser (808 nm, 1.8 W cm^−2^, 5 min) and cultured in an incubator at 37 °C for 48 h. After the incubation, the supernatant was removed, and the precipitate was washed with sterile PBS and stained with 1% crystal violet solution for 20 min. The stained bacterial biofilm was dissolved with 80% ethanol and its OD value at 590 nm was measured to evaluate the inhibitory effect of each treatment group on the biofilm.

### 3.13. Biofilm Destruction

The 100 μL bacterial solution (10^8^ CFU mL^−1^) was inoculated into 96-well plate and cultured at 37 °C for 48 h to form an integrated biofilm. The upper culture medium was removed and G@CuS was added. It was irradiated with NIR laser (808 nm, 1.8 W cm^−2^, 5 min) in the irradiation group, and then it was recultured in a 37 °C incubator for 40 min. The remaining steps are the same as above.

### 3.14. Cell Culture

Human umbilical vein epidermal cells (*HUVECs*) and mouse fibroblasts (*L929s*) were used for all experiments. These cells were both cultured in DMEM containing 10% (*v*/*v*) FBS and 1% (*v*/*v*) penicillin−streptomycin and incubated at 37 °C with 5% CO_2_.

### 3.15. Cytotoxicity

MTT assay was used to evaluate the toxicity of G@CuS on *HUVECs* and *L929s*. Two kinds of cells were added to a 96-well plate (10^4^ cells per well) and cultured overnight, respectively. Then, different concentrations of G@CuS were added and incubated with cells for 24 h. The cells were stained with MTT solution for 4 h and dissolved with DMSO, and its OD value at 490 nm was measured to evaluate the toxicity of G@CuS to mammalian cells.

### 3.16. In Vitro Cell Migration

The *L929s* and G@CuS (0.75 mg mL^−1^) were inoculated in 6-well plate (5 × 10^5^ cells per well) and cultured in incubator for 24 h, and then the cell layer was scratched with 200 μL pipette tip to simulate the incisional wound. The cells were photographed at 0, 12, 24, 48 h later, and the cell mobility was calculated. Fresh medium was used as blank control.

### 3.17. Hematolysis Assay

Whole blood was extracted from the orbit of healthy Balb/c female mice and red blood cells were obtained by freeze centrifugation. The PBS solution containing 20% (*v*/*v*) red blood cells were mixed with different concentrations of G@CuS and cocultured at 37 °C for 2 h. The mixture was separated by freeze centrifugation and the OD value of the supernatant at 540 nm was measured to evaluate the hemolysis rate of G@CuS. A total of 1% TritonX-100 was used as the positive hemolysis control and PBS as the negative control.

### 3.18. Mice Wound Model of S. aureus Infection

Female Balb/c mice (6 weeks, ~20 g) were purchased from Changzhou Cavens Biological Technology Co, Ltd., and domesticated in the laboratory for 1 week. All animal experiments were carried out in accordance with current guidelines for the care of laboratory animals and were approved by the proper committee of Changzhou University. The mouse wound model was established according to previously published methods. Briefly, an oval full-thickness wound (8 mm × 6 mm) was constructed on the back of the mouse with a hole punch, and then 20 μL *S. aureus* solution (10^8^ CFU mL^−1^) was used to induce infection in the wound for 2 days.

### 3.19. Treatment of S. aureus-Infected Wounds

Mice were divided into 5 groups with 3 mice in each group: PBS, CuS, Gel, G@CuS, and G@CuS + IR. Each group was given 50 μL corresponding samples every day and stopped on the 5th day, and the wound healing and weights of the mice was recorded every day. Nine days later, the mice were killed to take the skin tissue from the wound for bacterial culture. Then hematoxylin–eosin (H&E) staining, Masson’s trichrome staining, Gram staining, and immunohistochemical CD31 staining were used to evaluate the effect of bacterial clearance and healing of wounds in each group.

### 3.20. Statistical Analysis

All date in this work were presented as mean values **±** standard deviation (S.D.) with *n* ≥ 3. Prism 7.0 (GraphPad software, La Jolla, CA, USA) was used for statistical analysis. Two-tailed Student’s *t*-test or one-way ANOVA was performed for significance analysis. A *p* < 0.05 was regarded statistically significant.

## 4. Conclusions

In this work, we constructed a nano-delivery system G@CuS nanogel, which used the gelatinase-responsive cleavage of Gel to release the CuS NDs antimicrobial agents. Combined with the wound repair effect of gelatin itself, the G@CuS nanogel effectively treated wound bacterial infection and promoted wound healing. G@CuS exerted the phototherapy performance of CuS NDs under the irradiation of the NIR laser, producing ROS and heat, causing irreversible damage to and killing the bacteria. Meanwhile, the site-specific degradation of the Gel in the infected site effectively limited the oxidative stress toxicity of the CuS to the normal cells, increased the wound contraction rate, and showed excellent therapeutic potential in the mice wound model of *S. aureus* infection. Therefore, this work was expected to promote the further development of MNPs in biomedicine and provided solutions to prevent the development of drug resistance.

## Data Availability

Not applicable.

## Supplementary Materials

The following supporting information can be downloaded at: https://www.mdpi.com/article/10.3390/gels8070397/s1, Figure S1: Encapsulation efficiency; Figure S2: Particle sizes; Figure S3: Photothermal images; Figure S4: In vitro antibacterial activity of the CuS NDs; Figure S5: Standard curve of CuS NDs; Figure S6: In vitro antibacterial activity of the G@CuS; Figure S7: Anti-biofilm activity; Figure S8: Images of red blood cells; Figure S9: Effects of NIR irradiation on biocompatibility; Figure S10: Cells proliferation and migration; Figure S11: H&E staining images of major organs of mice.

## References

[1] Dąbrowska A.K., Spano F., Derler S., Adlhart C., Spencer N.D., Rossi R.M. (2018). The relationship between skin function, barrier properties, and body-dependent factors. Skin Res. Technol..

[2] Grice E.A., Kong H.H., Conlan S., Deming C.B., Davis J., Young A.C., Bouffard G.G., Blakesley R.W., Murray P.R., Green E.D. (2009). Topographical and temporal diversity of the human skin microbiome. Science.

[3] Wang X., Qiu L., Wang C., Gao Z., Zhou S., Cui P., Jiang P., Hu H., Ni X., Du X. (2022). Nanodot-doped peptide hydrogels for antibacterial phototherapy and wound healing. Biomater. Sci..

[4] Willyard C. (2018). Unlocking the secrets of scar-free skin healing. Nature.

[5] Wang J., Chen X., Zhao Y., Yang Y., Wang W., Wu C., Yang B., Zhang Z., Zhang L., Liu Y. (2019). pH-switchable antimicrobial nanofiber networks of hydrogel eradicate biofilm and rescue stalled healing in chronic wounds. ACS Nano.

[6] Malic S., Hill K.E., Playle R., Thomas D.W., Williams D.W. (2011). In vitro interaction of chronic wound bacteria in biofilms. J. Wound Care.

[7] Feng Y., Coradi Tonon C., Ashraf S., Hasan T. (2021). Photodynamic and antibiotic therapy in combination against bacterial infections: Efficacy, determinants, mechanisms and future perspectives. Adv. Drug Deliv. Rev..

[8] Duraão P., Balbontín R., Gordo I. (2018). Evolutionary mechanisms shaping the maintenance of antibiotic resistance. Trends Microbiol..

[9] Haghniaz R., Rabbani A., Vajhadin F., Khan T., Kousar R., Khan A.R., Montazerian H., Iqbal J., Libanori A., Kim H.J. (2021). Anti-bacterial and wound healing-promoting effects of zinc ferrite nanoparticles. J. Nanobiotechnol..

[10] Godoy-Gallardo M., Eckhard U., Delgado L.M., Puente Y., Hoyos-Nogués M., Gil F.J., Perez R.A. (2021). Antibacterial approaches in tissue engineering using metal ions and nanoparticles: From mechanisms to applications. Bioact. Mater..

[11] Wang C., Hong T., Cui P., Wang J., Xia J. (2021). Antimicrobial peptides towards clinical application: Delivery and formulation. Adv. Drug Deliv. Rev..

[12] Hancock R.E., Sahl H.G. (2006). Antimicrobial and host-defense peptides as new anti-infective therapeutic strategies. Nat. Biotechnol..

[13] Huo J., Jia Q., Huang H., Zhang J., Li P., Dong X., Huang W. (2021). Emerging photothermal-derived multimodal synergistic therapy in combating bacterial infections. Chem. Soc. Rev..

[14] Wang Y., Jin Y., Chen W., Wang J., Chen H., Sun L., Li X., Ji J., Yu Q., Shen L. (2019). Construction of nanomaterials with targeting phototherapy properties to inhibit resistant bacteria and biofilm infections. Chem. Eng. J..

[15] Xu J.W., Yao K., Xu Z.K. (2019). Nanomaterials with a photothermal effect for antibacterial activities: An overview. Nanoscale.

[16] Wang J., Wu H., Yang Y., Yan R., Zhao Y., Wang Y., Chen A., Shao S., Jiang P., Li Y.Q. (2017). Bacterial species-identifiable magnetic nanosystems for early sepsis diagnosis and extracorporeal photodynamic blood disinfection. Nanoscale.

[17] Liu W.Z., Zhang Y.X., You W.W., Su J.Q., Yu S.H., Dai T., Huang Y.M., Chen X.Y., Song X.R., Chen Z. (2020). Near-infrared-excited upconversion photodynamic therapy of extensively drug-resistant acinetobacter baumannii based on lanthanide nano-particles. Nanoscale.

[18] Feng Z., Liu X., Tan L., Cui Z., Yang X., Li Z., Zheng Y., Yeung K.W.K., Wu S. (2018). Electrophoretic deposited stable chitosan@MoS_2_ coating with rapid in situ bacteria-killing ability under dual-light irradiation. Small.

[19] Curcio A., Walle A., Benassai E., Serrano A., Luciani N., Menguy N., Manshian B.B., Sargsian A., Soenen S., Espinosa A. (2021). Massive intracellular remodeling of CuS nanomaterials produces nontoxic bioengineered structures with preserved photothermal potential. ACS Nano.

[20] Wang S., Riedinger A., Li H., Fu C., Liu H., Li L., Liu T., Tan L., Barthel M.J., Pugliese G. (2015). Plasmonic copper sulfide nanocrystals exhibiting near-infrared photothermal and photodynamic therapeutic effects. ACS Nano.

[21] Li L., Rashidi L.H., Yao M., Ma L., Chen L., Zhang J., Zhang Y., Chen W. (2017). CuS nanoagents for photodynamic and photothermal therapies: Phenomena and possible mechanisms. Photodiagn. Photodyn. Ther..

[22] Zhou L.Q., Chen F., Hou Z.S., Chen Y.W., Luo X.L. (2021). Injectable self-healing CuS nanoparticle complex hydrogels with antibacterial, anti-cancer, and wound healing properties. Chem. Eng. J..

[23] Xu C., Akakuru O.U., Ma X., Zheng J., Zheng J., Wu A. (2020). Nanoparticle-based wound dressing: Recent progress in the detection and therapy of bacterial infections. Bioconjug. Chem..

[24] Li Q., Wang W., Feng H., Cao L., Wang H., Wang D., Chen S. (2021). NIR-triggered photocatalytic and photothermal performance for sterilization based on copper sulfide nanoparticles anchored on Ti_3_C_2_T_x_ MXene. J. Colloid Interface Sci..

[25] Tao B., Lin C., Deng Y., Yuan Z., Shen X., Chen M., He Y., Peng Z., Hu Y., Cai K. (2019). Copper-nanoparticle-embedded hydrogel for killing bacteria and promoting wound healing with photothermal therapy. J. Mater. Chem. B..

[26] Kornblatt A.P., Nicoletti V.G., Travaglia A. (2016). The neglected role of copper ions in wound healing. J. Inorg. Biochem..

[27] Zhou W., Zi L., Cen Y., You C., Tian M. (2020). Copper sulfide nanoparticles-incorporated hyaluronic acid injectable hydrogel with enhanced angiogenesis to promote wound healing. Front. Bioeng. Biotechnol..

[28] Wu C., Zhou Y., Xu M., Han P., Chen L., Chang J., Xiao Y. (2013). Copper-containing mesoporous bioactive glass scaffolds with multifunctional properties of angiogenesis capacity, osteostimulation and antibacterial activity. Biomaterials.

[29] Qiao Y., Ping Y., Zhang H., Zhou B., Liu F., Yu Y., Xie T., Li W., Zhong D., Zhang Y. (2019). Laser-activatable CuS nanodots to treat-multidrug-resistant bacteria and release copper ion to accelerate healing of infected chronic nonhealing wounds. ACS Appl. Mater. Interfaces.

[30] Forsyth P.A., Wong H., Laing T.D., Rewcastle N.B., Morris D.G., Muzik H., Leco K.J., Johnston R.N., Brasher P.M., Sutherland G. (1999). Gelatinase-A (MMP-2), gelatinase-B (MMP-9) and membrane type matrix metalloproteinase-1 (MT1-MMP) are involved in different aspects of the pathophysiology of malignant gliomas. Br. J. Cancer.

[31] Young S., Wong M., Tabata Y., Mikos A.G. (2005). Gelatin as a delivery vehicle for the controlled release of bioactive molecules. J. Control Release.

[32] Elzoghby A.O. (2013). Gelatin-based nanoparticles as drug and gene delivery systems reviewing three decades of research. J. Control Release.

[33] Ansari M.M., Ahmad A., Kumar A., Alam P., Khan T.H., Jayamurugan G., Raza S.S., Khan R. (2021). Aminocellulose-grafted-polycaprolactone coated gelatin nanoparticles alleviate inflammation in rheumatoid arthritis: A combinational therapeutic approach. Carbohydr. Polym..

[34] Diba M., Koons G.L., Bedell M.L., Mikos A.G. (2021). 3D printed colloidal biomaterials based on photo-reactive gelatin nanoparticles. Biomaterials.

[35] Li L.L., Xu J.H., Qi G.B., Zhao X., Yu F., Wang H. (2014). Core-shell supramolecular gelatin nanoparticles for adaptive and “on-demand” antibiotic delivery. ACS Nano.

[36] Lei X., Qiu L., Lan M., Du X., Zhou S., Cui P., Zheng R., Jiang P., Wang J., Xia J. (2020). Antibacterial photodynamic peptides for staphylococcal skin infection. Biomater. Sci..

[37] Mao L., Wang L., Zhang M., Ullah M.W., Liu L., Zhao W., Li Y., Ahmed A.A.Q., Cheng H., Shi Z. (2021). In situ synthesized selenium nanoparticles-decorated bacterial cellulose/gelatin hydrogel with enhanced antibacterial, antioxidant, and anti-inflammatory capabilities for facilitating skin wound healing. Adv. Healthc. Mater..

[38] Zhang X., Liu W., Yang D., Qiu X. (2019). Biomimetic supertough and strong biodegradable polymeric materials with improved thermal properties and excellent UV-blocking performance. Adv. Funct. Mater..

[39] Zhang X.H., Liu M.H., Kang Z.W., Wang B.Q., Wang B., Jiang F.Y., Wang X.S., Yang D.P., Luque R. (2020). NIR-triggered photocatalytic/photothermal/photodynamic water remediation using eggshell-derived CaCO_3_/CuS nanocomposites. Chem. Eng. J..

[40] Fu J.J., Zhang J.Y., Li S.P., Zhang L.M., Lin Z.X., Liang L., Qin A.P., Yu X.Y. (2018). CuS nanodot-loaded thermosensitive hydrogel for anticancer photothermal therapy. Mol. Pharm..

[41] Lin A., Liu Y., Zhu X., Chen X., Liu J., Zhou Y., Qin X., Liu J. (2019). Bacteria-responsive biomimetic selenium nanosystem for multidrug-resistant bacterial infection detection and inhibition. ACS Nano.

[42] Badawi N., El-Say K., Attia D., El-Nabarawi M., Elmazar M., Teaima M. (2020). Development of pomegranate extract-loaded solid lipid nanoparticles: Quality by design approach to screen the variables affecting the quality attributes and characterization. ACS Omega.

