# Supplementary material for: Gelatinase Responsive Nanogel for Antibacterial Phototherapy and Wound Healing
Source: Gels. 2022 Jun 23;8(7):397. doi: 10.3390/gels8070397 (PMC9320007; doi:10.3390/gels8070397)
Supplement: Supplementary file 1 [file gels-08-00397-s001.zip › gels-1723559-supplementary.pdf]

gels

Article

Gelatinase Responsive Nanogel for Antibacterial Phototherapy and Wound Healing

Qianqian Han [1,†], Xuan Wang [1,†], Lin Qiu [1,†], Xinpei Zhou [1], Zexuan Hui [1], Xinye Ni [2,*], Yang Xuan [3], Xiaoling Lei [4] and Jianhao Wang [1,*]

1 School of Pharmacy, Changzhou University, Changzhou 213164, China; 200817z1018@smail.cczu.edu.cn (Q.H.); 19085235788@smail.cczu.edu.cn (X.W.); linqiu@cczu.edu.cn (L.Q.); 19479125@smail.cczu.edu.cn (X.Z.); 19479127@smail.cczu.edu.cn (Z.H.)
2 The Affiliated Changzhou No. 2 People's Hospital of Nanjing Medical University, Changzhou 213003, China
3 Key Lab of Biotechnology and Bioresources Utilization of Ministry of Education, College of Life Science, Dalian Minzu University, Dalian 116600, China; xuanyang@dlnu.edu.cn
4 College of Life Science and Technology, Huazhong University of Science and Technology, Wuhan 430074, China; d202180741@hust.edu.cn
* Correspondence: nxy@njmu.edu.cn (X.N.); minuswan@cczu.edu.cn. (J.W.)
† These authors contributed equally to this work.

Supplementary Materials

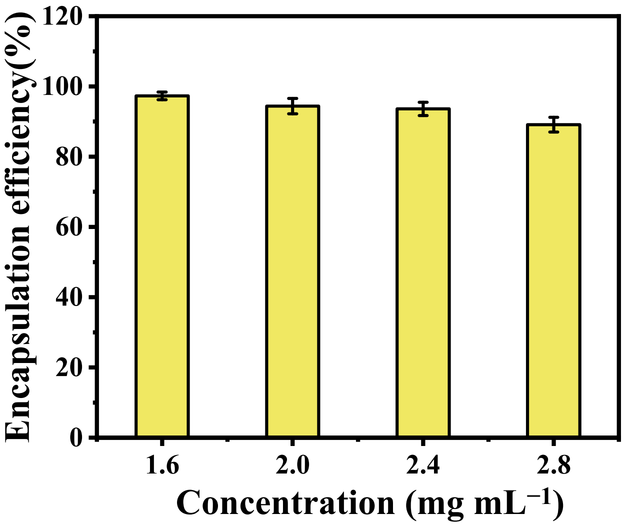

Figure S1. Encapsulation efficiency (EE) of CuS NDs in Gel.

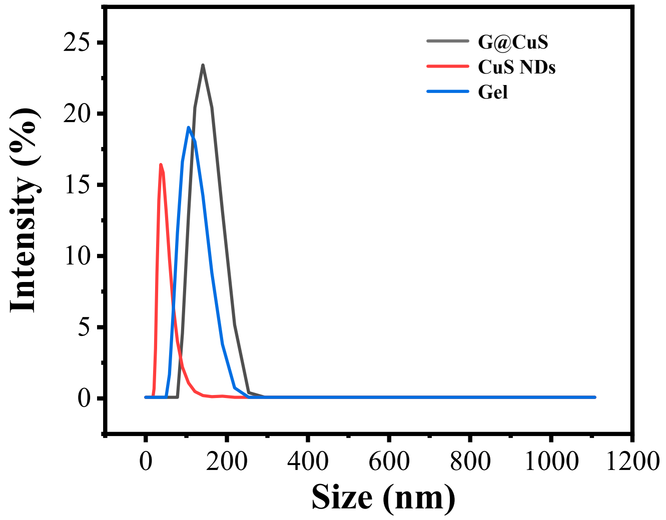

Figure S2. Particle sizes of the G@CuS, CuS NDs and Gel.

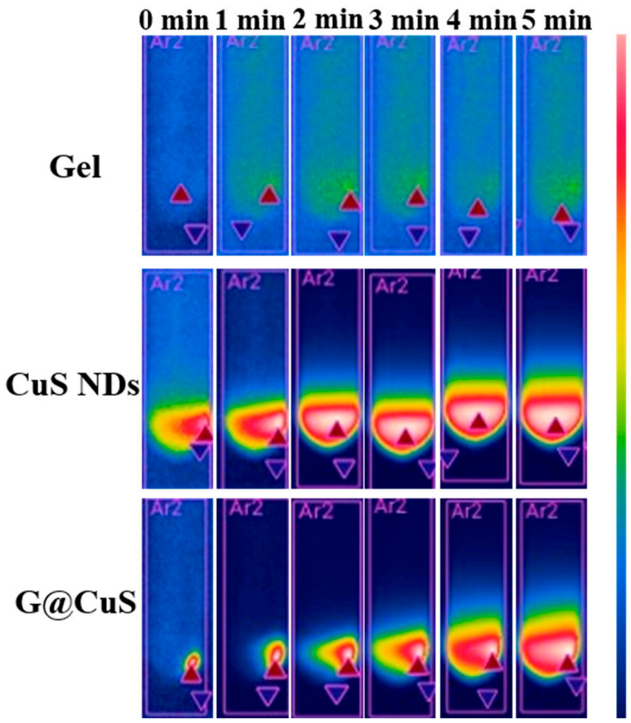

Figure S3. Photothermal images of Gel, CuS NDs and G@CuS under NIR (808 nm, 1.8 W cm^{-2}) irradiation.

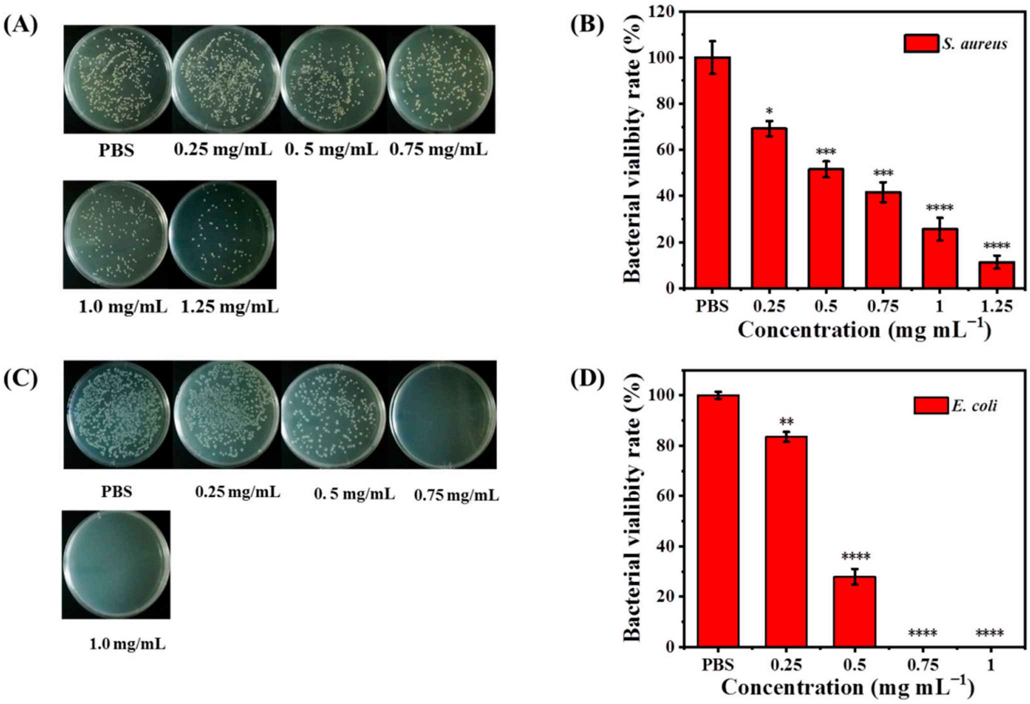

Figure S4. *In vitro* antibacterial activity of the CuS NDs. Colony images (**A**) and a statistical chart (**B**) of *S. aureus* incubated with different concentrations of the CuS NDs. Colony images (**C**) and a statistical chart (**D**) of *E. coli* incubated with different concentrations of the CuS NDs. (n=3; *p <0.05, **p < 0.01, ***p < 0.001, ****p < 0.0001).

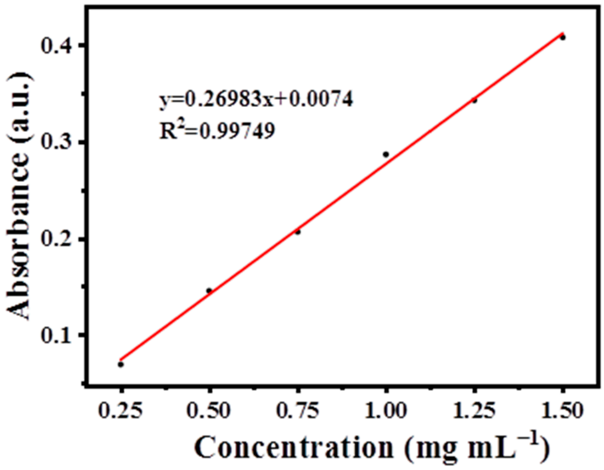

Figure S5. The linear calibration curve between the CuS NDs concentration and its absorbance.

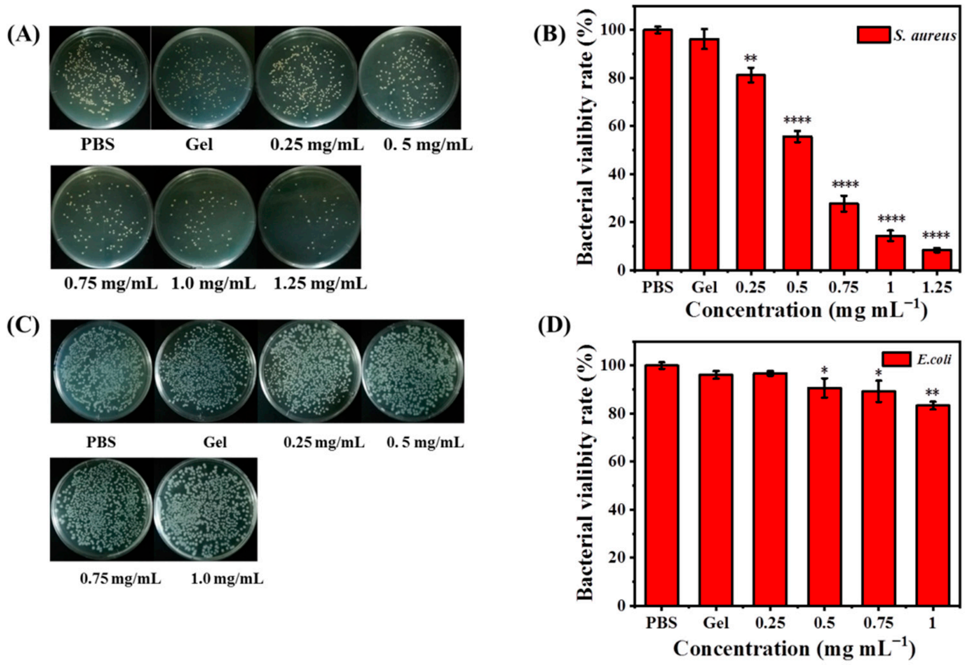

Figure S6. *In vitro* antibacterial activity of the G@CuS. Colony images (**A**) and a statistical chart (**B**) of *S. aureus* incubated with different concentrations of the G@CuS. Colony images (**C**) and a statistical chart (**D**) of *E. coli* incubated with different concentrations of the G@CuS. (n=3; *p <0.05, **p < 0.01, ****p < 0.0001).

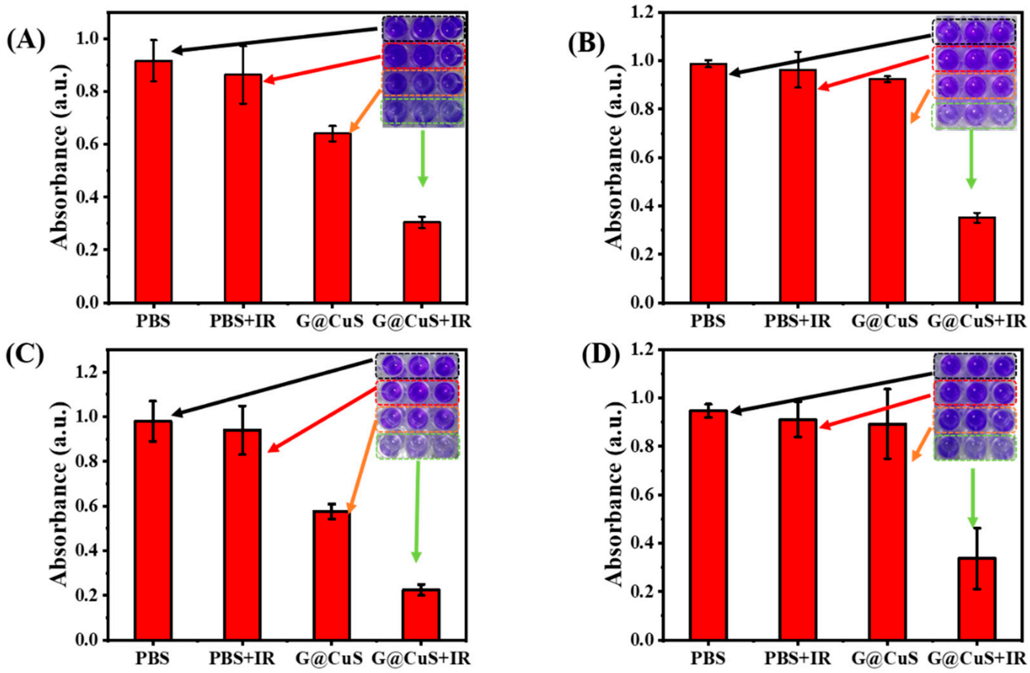

Figure S7. Anti-biofilm activity of the G@CuS before and after NIR laser (808 nm, 1.8 W cm^{-2}, 5 min) irradiation. Crystal violet staining images and its corresponding absorbance for inhibiting *S. aureus* (**A**) and *E. coli* (**B**) biofilms before and after NIR laser irradiation. Crystal violet staining images and its corresponding absorbance for destroying *S. aureus* (**C**) and *E. coli* (**D**) biofilms before and after NIR laser irradiation.

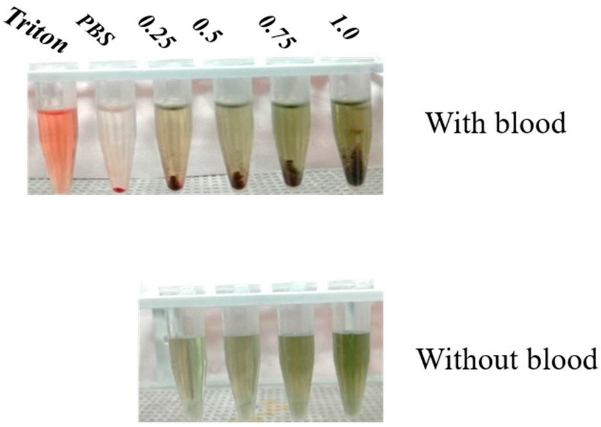

Figure S8. Images of red blood cells incubated with different concentrations of the CuS NDs.

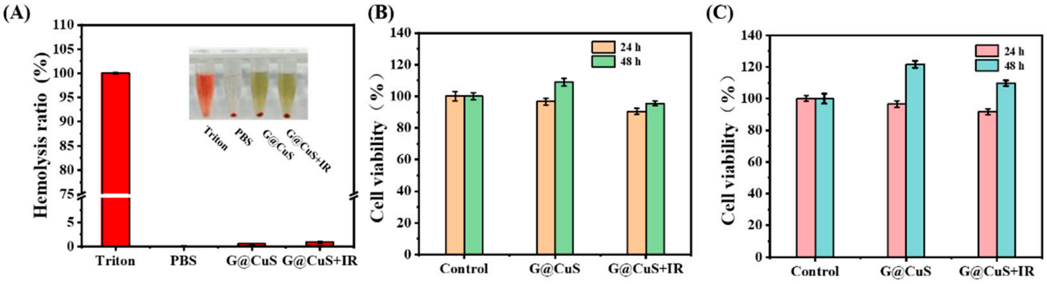

Figure S9. Effects of NIR (808 nm, 1.8 W cm^{-2}, 5min) irradiation on biocompatibility. (**A**) Hemolysis ratio and images of red blood cells incubated with the G@CuS (0.75 mg mL^{-1}) before and after NIR laser (808 nm, 1.8 W cm^{-2}, 5 min) irradiation. Cytotoxicity of the G@CuS (0.75 mg mL^{-1}) incubated with *L929s* (**B**) and *HUVECs* (**C**) before and after NIR laser (808 nm, 1.8 W cm^{-2}, 5 min) irradiation.

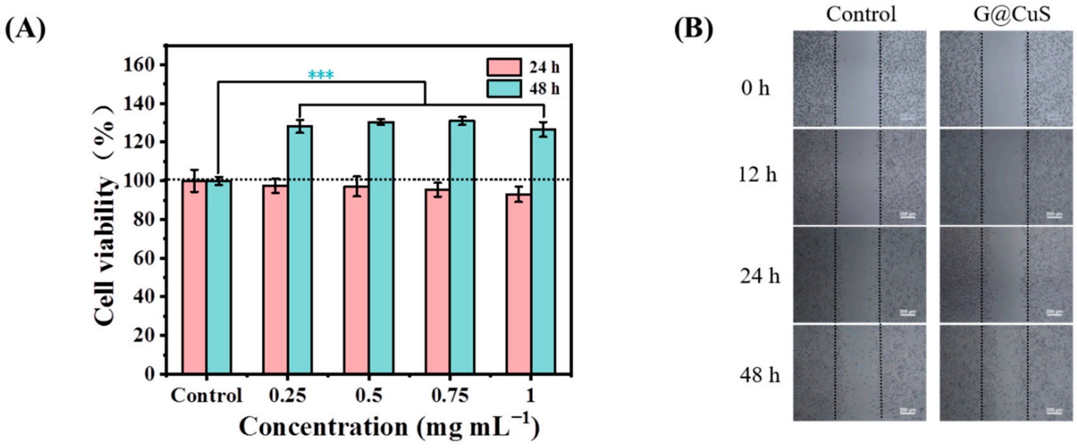

Figure S10. Promoting effect of G@CuS on cells proliferation and migration. (**A**) *L929s* viability after the treatment of G@CuS for 24 and 48 h. (**B**) Photographs of *L929s* migration at different times. (n=3; ***$p < 0.001$).

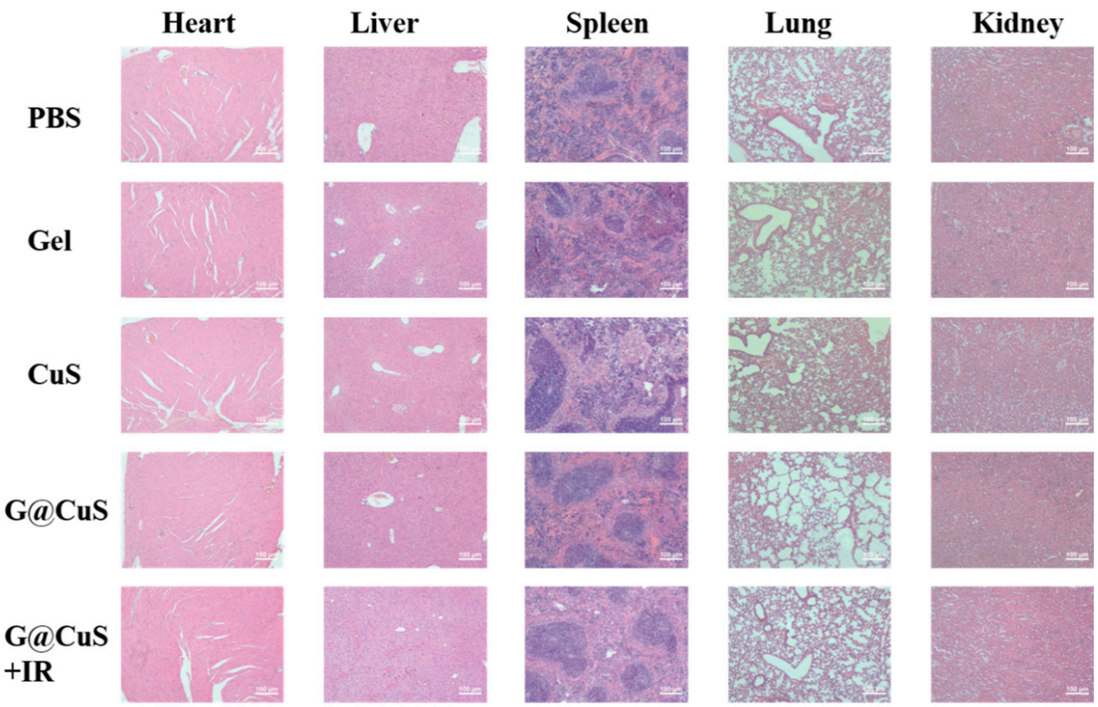

Figure S11. H&E staining images of major organs (heart, liver, spleen, lung and kidney) of mice in each treatment group.
